# Profiling microRNA expression in murine bone healing and non-union formation: Role of miR-140 during the early stage of bone healing

**DOI:** 10.1371/journal.pone.0218395

**Published:** 2019-07-19

**Authors:** Marcel Orth, Claudia Scheuer, Christina Backes, Andreas Keller, Mika F. Rollmann, Benedikt J. Braun, Nicole Ludwig, Eckart Meese, Tim Pohlemann, Matthias W. Laschke, Michael D. Menger, Tina Histing

**Affiliations:** 1 Department of Trauma, Hand and Reconstructive Surgery, Saarland University, Homburg, Germany; 2 Institute for Clinical and Experimental Surgery, Saarland University, Homburg, Germany; 3 Department of Clinical Bioinformatics, Saarland University, Saarbrücken, Germany; 4 Department of Human Genetics, Saarland University, Homburg, Germany; Augusta University, UNITED STATES

## Abstract

Although cellular and molecular mechanisms during the course of bone healing have been thoroughly investigated, the regulation of gene expression by microRNA during bone regeneration is still poorly understood. We hypothesized that nonunion formation is associated with different microRNA expression patterns and that target proteins of these microRNAs are differently expressed in callus tissue of nonunions compared to physiologically healing bones. In a well-established femoral osteotomy model in *CD-1* mice osteotomies were induced which result either in healing or in nonunion formation. MicroRNA and target protein expression was evaluated by microarray, quantitative real-time polymerase chain reaction (qrt-PCR) and Western blot. Microarray analyses demonstrated 44 microRNAs to be relevant for nonunion formation compared to physiological bone healing. In nonunions qrt-PCR could validate a higher expression of microRNA-140-3p and microRNA-140-5p. This was associated with a reduced expression of Dnpep and stromal cell-derived factor (SDF)-1α, which are both known to be target proteins of microRNA-140 and also to be involved in the process of bone healing. These data suggest that an increased expression of microRNA-140-3p and microRNA-140-5p markedly contributes to the development of nonunions, most probably by affecting bone morphogenetic protein (BMP)-2 function during the early stage of healing due to a reduced SDF-1α expression.

## 1. Introduction

Approximately 5–10% of all fractures show delayed bone healing or nonunion formation [1]. Bone healing is a highly complex and well-orchestrated process, which involves numerous regulating factors. It is dependent on the interaction of bone marrow-derived hematopoietic and immune cells with endothelial and skeletal progenitor cells from the blood stream and the surrounding tissues [2]. By means of in vivo and in vitro experiments, considerable efforts have been made to understand the cellular and molecular mechanisms, which contribute to the process of bone healing [2,3]. Accordingly, the knowledge on distinct cellular and biochemical pathways during bone healing has markedly increased during the last two decades. In contrast, the regulation of gene expression during bone regeneration is still poorly understood.

The recent discovery of microRNA (miR) revealed a novel form of regulating gene expression. MiRs are small non-coding ribonucleic acid (RNA) molecules that are believed to regulate one third of the genes in the genome [4]. MiRs have been linked to many diseases and are currently pursued as clinical diagnostic and therapeutic targets [4]. In short, miR therapies have the advantages to circumvent secondary surgery and provide an ‘off-the-shelf’ availability, making them promising treatment strategies also for bone repair.

The regulating effect of miRs is mediated by specific binding of messenger RNA (mRNA), thereby causing its degradation with subsequent inhibition of protein expression [5]. However, the effect of miRs is not only a simple ‘on-off’ mechanism, but rather causes a decrease of mRNA expression [5]. Besides, miR interactions can also increase the expression of proteins as demonstrated for miR-373 that promotes the expression of E-cadherin by enhanced promoter occupancy due to RNA polymerase II [6]. Moreover, it has been reported that miRs are tissue-specific such as e.g. miR-122 for the liver [7]. Accordingly, the effects of miRs are highly complex and significantly contribute to the proteome of cells, and, by these means, to the function of individual organs.

Several miRs are thought to be involved in the process of bone healing. Inhibition of miR-92a has been reported to enhance fracture healing by promoting angiogenesis [8]. Other miRs such as e.g. miR-20a, miR-29b, miR-2861, miR-138, miR-26a, and miR-21 have been shown to mediate regulatory effects for osteoblastic differentiation [9].

Based upon these previous findings and the regulatory role of miRs, we hypothesize that (i) nonunion formation exerts a different miR expression profile compared to physiological bone healing and that (ii) target proteins of differently expressed miRs show a different expression within the callus tissue of nonunions compared to physiologically healing bones. To test these hypotheses, we induced bone healing and nonunion formation in femurs of mice and studied the expression of miRs and their target proteins within the callus tissue using microarray, quantitative real-time polymerase chain reaction (qrt-PCR) and Western blot analysis.

## 2. Materials and methods

### 2.1 Animals

In the present study, 14 male and female *CD-1* mice (mean body weight: 30–40 g; age: 9–14 weeks) were used. The animals were bred at the animal facility of the Institute for Clinical and Experimental Surgery, Saarland University, Germany, and were housed at a regular 12 hour / 12 hour light and dark cycle with free access to tap water and standard pellet food (Altromin, Lage, Germany). The animals were randomly distributed to the two groups. The study was conducted in accordance with the German legislation on protection of animals and the NIH Guidelines for the Care and Use of Laboratory Animals and has been approved by the animal protection committee of the Office for Consumer Protection, Department Food Administration and Veterinary Services, Saarland, Saarbrücken, Germany (approval no. VV 53/2013).

### 2.2 Surgical procedure

To study the expression profile of miRs and their target proteins during physiological bone healing and nonunion formation, a well-established femoral osteotomy model was used [10,11]. In brief, for anesthesia the mice received an intraperitoneal injection of xylazine (25 mg / kg body weight; Rompun; Bayer, Leverkusen, Germany) and ketamine (75 mg / kg body weight; Pharmacia GmbH, Erlangen, Germany). The right knee was opened via a ~4 mm medial parapatellar incision to dislocate the patella to the lateral side. Then, a distally flattened 24 Gauge needle with a diameter of 0.55 mm was retrogradely inserted into the intramedullary canal through a 0.5 mm drill hole at the intercondylar notch. The needle served as an intramedullary pin. The needle was flattened at its distal end to prevent its dislocation during the healing process. Then, the diaphysis of the femur was laterally accessed and a custom-made clip with a length of 6 mm was ventro-dorsally implanted into the bone. Finally, an osteotomy with a gap size of 0.25 mm was performed in the union group (Union; n = 7). Previous studies could demonstrate that this gap size promotes physiological bone healing [10,12]. In the nonunion group (Nonunion; n = 7) a gap size of 1.8 mm was created. In addition, the periosteum was stripped 2 mm proximally and distally of the gap along the longitudinal axis of the femoral bone. The metallic clip inserted before creation of the gap guaranteed that the gap size was maintained. Previous studies have shown that this results in nonunion formation [11,13]. Wound closure completed the surgical procedure.

On day 7, secondary dislocation of the metallic implants was excluded by X-rays of the operated femurs. Thereafter, the mice were sacrificed by an overdose of barbiturates. Callus tissue was harvested and transferred to RNA*later* stabilization reagent (n = 4 each group; Qiagen, Hilden, Germany) at 4 °C for 3 days or protein lysis buffer (n = 3 each group). Samples were stored at -80 °C until further use.

### 2.3 Isolation of total cellular RNA including miR

The frozen specimens stored in RNA*later* stabilization reagent (Qiagen) were used for RNA extraction by means of the guanidinium thiocyanate-phenol-chloroform extraction method [14]. For this purpose, 700 μL QIAzol lysis reagent (Qiagen) were added and tissues were homogenized mechanically (Miccra-D1, Miccra GmbH, Heitersheim, Germany) for 30 seconds. After incubation for 5 minutes at room temperature, 140 μL chloroform (Carl Roth GmbH, Karlsruhe, Germany) was added and incubated for 3 minutes at room temperature. Next, the specimens were centrifuged at 4 °C at 12,000 xg for 15 minutes. The RNA-containing alcohol purified superior phase was used for processing miR with the miRNeasy Mini Kit (Qiagen) following the manufacturer’s instructions. The obtained extracts, which were purified by means of several column spin centrifugation steps, was quantified by RNA concentration measuring using the Nanodrop2000 (ThermoScientific, Thermo Fisher Scientific, Waltham, USA). RNA quality was controlled using the Bioanalyzer2100 and the Agilent Small RNA Kit (Agilent, Frankfurt, Germany). These total RNA extracts including miR were then used for the miR microarray or the qrt-PCR analyses.

### 2.4 MiR microarray

Microarray analyses were performed according to manufacturer’s instructions using SurePrint G3 8 x 60K miR microarrays (Agilent, Santa Clara, USA), as described previously [15,16]. This profiling method contained 40 replicates of each murine miR listed in miRBase at the time of investigation. In brief, a total of 100 ng total RNA was used as input to generate Cy3 labelled RNA using the Complete Labeling and Hyb Kit (Agilent) according to manufacturer’s instructions. In detail, RNA was dephosphorylated using Calf Intestinal Alkaline Phosphatase, subsequently denaturated and labelled using Cy3-pCp for 2 hours. The Cy3-labelled RNA was dried under vaccuum, resuspended in the hybridization mixture and applied to the microarray for hybridization for 20 hours at 55 °C and 20 RPM. The next day, microarrays were washed using Gene Expression Wash Buffer Kit (Agilent), air dried and then scanned using Agilent microarray scanner with 3 μm resolution in double pass mode. Raw expression values for the miRs were extracted using Agilent Feature extraction software. MiRs with significant differences in its expression and / or a mean fold change (FC) of either ≥2.0 or ≤-2.0 between the Union and Nonunion group were defined as relevant miRs. This FC threshold was chosen upon previous studies investigating miR expression during bone healing [17,18].

### 2.5 Qrt-PCR

For further qrt-PCR analyses, 4 relevant miRs were selected based upon the microarray results. These microRNAs were chosen either because of a high fold change (mmu-miR-5099), a highly significant difference (mmu-miR-3096-5p) and based upon an extensive literature research (mmu-miR-140-3p and mmu-miR-140-5p) [18–22]. To compare the expression level of the selected miRs between the two study groups, mature miR was converted into complementary DNA (cDNA). For this purpose, the miScript II RT Kit PCR System (Qiagen) was used according to the manufacturer’s instructions. In brief, polyadenylation and reverse transcription reaction were performed with a mixture of poly-(A) polymerase and reverse transcriptase, using oligo-dT primers as a one-step method. The generated cDNA served as input in the subsequent qrt-PCR using the miScript SYBR Green PCR Kit and miScript Primer assays sets (Qiagen). Quantification of cDNA and data analyses were conducted with the MiniOpticon real time PCR system as well as the CFX Manager software (BioRad, Munich, Germany). Expression of miR levels were normalized to a murine nucleolar RNA housekeeping gene (SNORD61-11 miScript Primer Assay, Qiagen). All reactions were performed in duplicates, and mean cycle quantity (Cq) values of the duplicates were used to calculate the ΔΔ Gene expression rate [Cq] for each miR compared with the housekeeping control gene in each sample.

### 2.6 Western blot

Western blot analyses were performed to study protein expression within the callus tissue. The analyses included the assessment of the expression of stromal cell-derived factor (SDF)-1α and aspartyl aminopeptidase Dnpep, which have both been reported to be regulated by miR-140 [19,23]. After saving the whole-protein fraction, proteins were separated and transferred to membranes by standard protocols. Proteins were then probed using anti-SDF-1α (sc-74271, 1:50; Santa Cruz Biotechnology, Heidelberg, Germany) and anti-Dnpep (1:50; Abcam, Cambrigde, UK) antibodies. The appropriate peroxidase-conjugated anti-IgG antibodies served as secondary antibodies (1:1500; Dako, Hamburg, Germany and 1:1000; R&D Systems, Wiesbaden, Germany). To visualize protein expression, luminol-enhanced chemiluminescence was performed after exposure of the membrane to the Intas ECL Chemocam Imager (Intas Science Imaging Instrument GmbH, Göttingen, Germany). Detected signals were normalized to β-actin signals (1:5000; Sigma-Aldrich, Taufkirchen, Germany) to correct for unequal loading.

### 2.7 Statistical analyses

For the microarray analyses, the 40 replicates of each miR from the arrays were merged to one expression value. The miR expression values were log2 transformed and quantile normalized [24] to account for inter-array effects using the statistical software R with the preprocessCore package. All further analyses were performed on the normalized intensity values. For hierarchical cluster analysis, the Heatplus package (Bioconductor, open source) was used. The statistical analyses of the microarray results were performed only for miRs that could be detected in all samples (signal over background).

Data of qrt-PCR and Western blot analyses are given as means ± standard error of the mean (SEM). Data were first tested for normal distribution and equal variance. Comparison between the experimental groups was performed by the Student’s *t*-test. The statistical analyses for qrt-PCR and Western blot analyses were performed using the SigmaPlot software 13.0 (Systat Software, Erkrath, Germany).

A *p*-value <0.05 for all statistical analyses was considered to indicate significant differences.

## 3. Results

### 3.1 Radiological analyses

**Fig 1:**
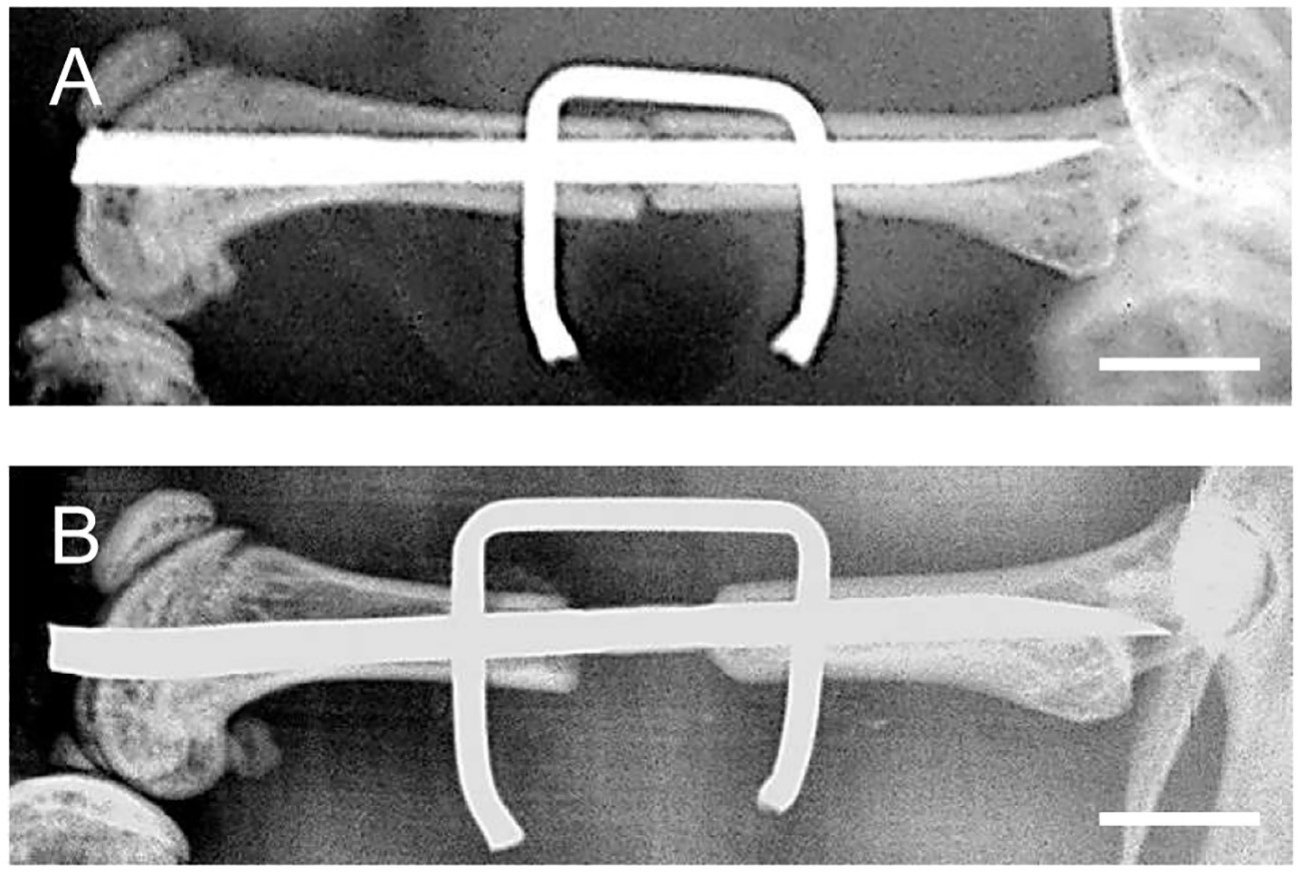
Radiological analyses of osteotomized femurs at day 7 after surgery. **A, B**: X-rays of the osteotomized mouse femurs with a segmental defect of 0.25 mm (**A**) or 1.8mm (**B**) stabilized by the ‘pin-clip’ technique at 7 days after surgery. The bone fragments in the Union group (**A**) have direct contact, whereas bone fragments of the Nonunion group (**B**) presented with a persistent gap. As expected, no radiological signs of osseous bridging can be observed at this early 7-day time point. Images do not show signs of implant dislocation. Bars represent 2 mm.

Representative X-rays showed direct contact of the bone fragments in the Union group (Fig 1A), whereas the Nonunion group presented with a persistent gap between the bone fragments (Fig 1B). As expected, none of the animals showed signs of osseous bridging at this early 7-day time point. Implant dislocations were not observed.

### 3.2 Microarray analyses

**Fig 2:**
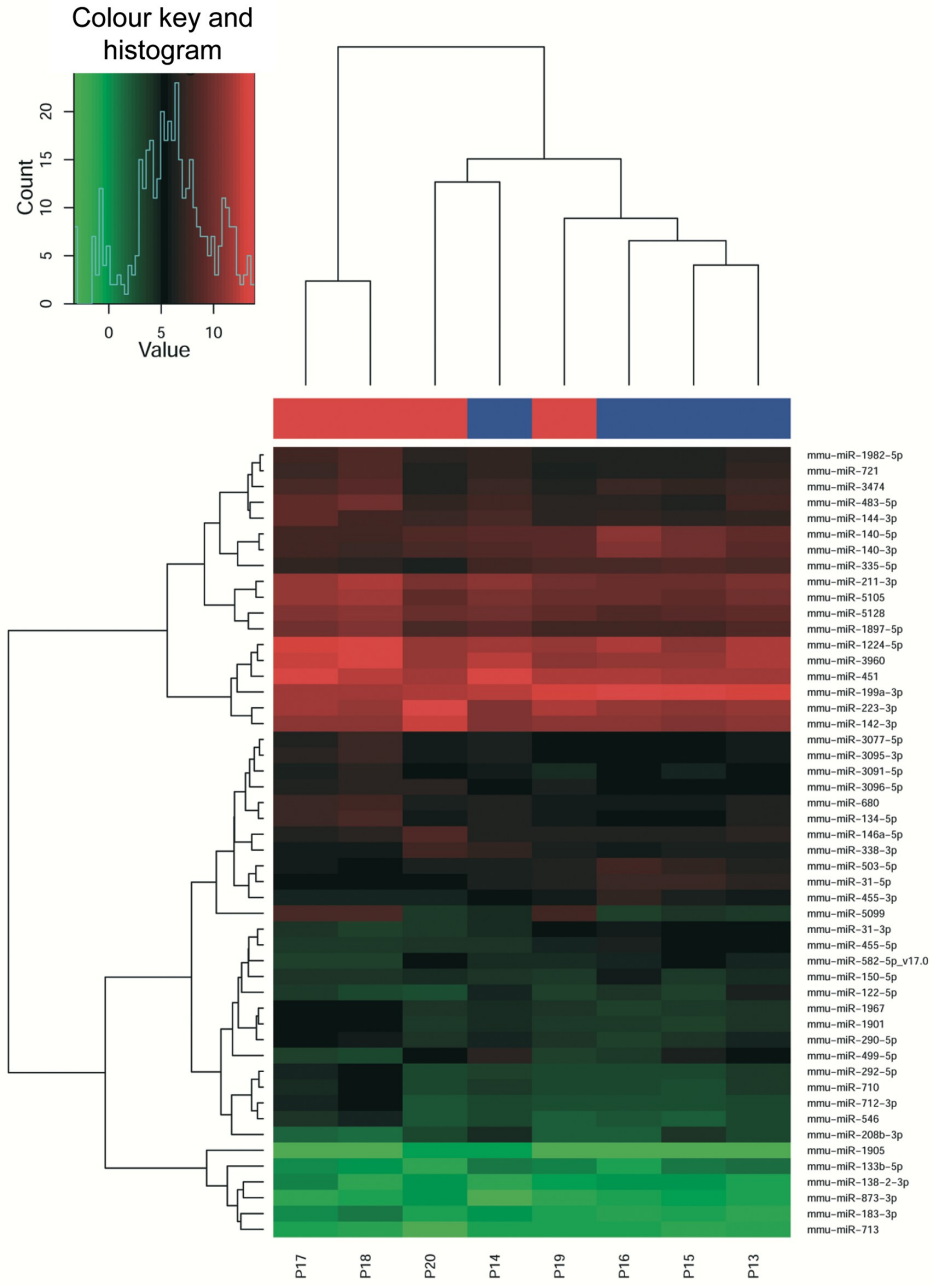
Microarray analyses of callus tissue reveals a typical miR pattern for each group. Heatmap using the 50 miRs with the highest variance over all samples analyzed at 7 days after osteotomy. Specimens (columns; P13—P20) and miRs (rows) are indicated. Color key and histogram are given. The color of the bar under the dendrogram of the hierarchical cluster analysis indicates the tissue samples which will result in union (red) or in nonunion (blue) of the osteotomy.

Using miR microarray analyses a total of 1079 murine miRs (mmu-miR) were analyzed to detect differences in the miR expression profile between the two study groups. A number of 342 of these miRs could be detected in all samples and were, therefore, used for further statistical analyses. Hierarchical cluster analyses of the 50 miRs with the highest variance over all analyzed samples at 7 days after osteotomy revealed two main clusters, suggesting a typical miR pattern for each group (Fig 2). However, Euclidean distance showed proximity of two samples of the Union group (P19 and P20) to the samples of the Nonunion group at this early time point, as indicated by the dendrogram of the heatmap (Fig 2). Of interest, 44 of the miRs that could be detected in all samples, showed either significant differences in expression or a FC of either ≥2.0 or ≤-2.0 and were, therefore, considered to be relevant (Table 1). Only 5 miRs fulfilled both criteria. From all relevant miRs, the miR with the highest FC (mmu-miR-5099) and three significantly different miRs (mmu-miR-140-3p, mmu-miR-140-5p, mmu-miR-3096-5p) were selected for further qrt-PCR analyses.

**Table 1:** Microarray results.

miRNA	*p*	mean FC
**mmu-miR-3096-5p**	**0,001**	**-2,34**
mmu-miR-669n	0,002	-1,48
mmu-miR-24-2-5p	0,003	1,26
mmu-miR-5115	0,004	-1,79
mmu-miR-23a-3p	0,005	1,19
mmu-miR-511-3p	0,006	-1,48
mmu-miR-10b-5p	0,013	-1,32
mmu-miR-27a-3p	0,014	1,25
mmu-miR-669l-5p	0,015	-1,40
mmu-miR-5118	0,016	-1,89
mmu-miR-468-3p	0,016	-1,67
mmu-miR-132-3p	0,016	1,60
mmu-miR-24-1-5p	0,016	1,45
mmu-miR-351-5p	0,016	1,39
mmu-miR-342-5p	0,018	1,14
mmu-miR-32-3p	0,019	-1,59
mmu-miR-674-5p	0,021	1,23
**mmu-miR-31-5p**	**0,022**	**2,64**
mmu-miR-3099-3p	0,023	-1,75
mmu-miR-615-3p	0,024	1,32
mmu-miR-5107	0,026	-1,37
mmu-miR-214-5p	0,027	1,65
mmu-miR-181d-5p	0,031	1,60
**mmu-miR-31-3p**	**0,033**	**2,39**
mmu-miR-466i-5p	0,037	-1,48
mmu-let-7a-1-3p	0,039	1,17
mmu-miR-99b-3p	0,039	1,25
mmu-miR-199b-5p	0,041	1,60
mmu-miR-3470a	0,041	-1,86
mmu-miR-335-3p	0,042	1,62
mmu-miR-181b-5p	0,044	1,51
mmu-miR-3069-3p	0,047	1,23
mmu-miR-1196-5p	0,048	-1,30
mmu-miR-374c-5p	0,048	1,23
**mmu-miR-140-5p**	**0,048**	**2,70**
**mmu-miR-140-3p**	**0,049**	**2,63**
mmu-miR-5099	0,054	-8,83
mmu-miR-199a-3p	0,056	2,06
mmu-miR-335-5p	0,064	2,33
mmu-miR-455-3p	0,065	2,18
mmu-miR-503-5p	0,068	2,01
mmu-miR-455-5p	0,087	2,03
mmu-miR-223-3p	0,133	-2,22
mmu-miR-483-5p	0,291	-2,01

Overview of miRs with either significant differences and / or FC of ≥2 or ≤-2 between the groups Union and Nonunion. MiRs fulfilling both criteria are highlighted.

### 3.3 Qrt-PCR analyses

**Fig 3:**
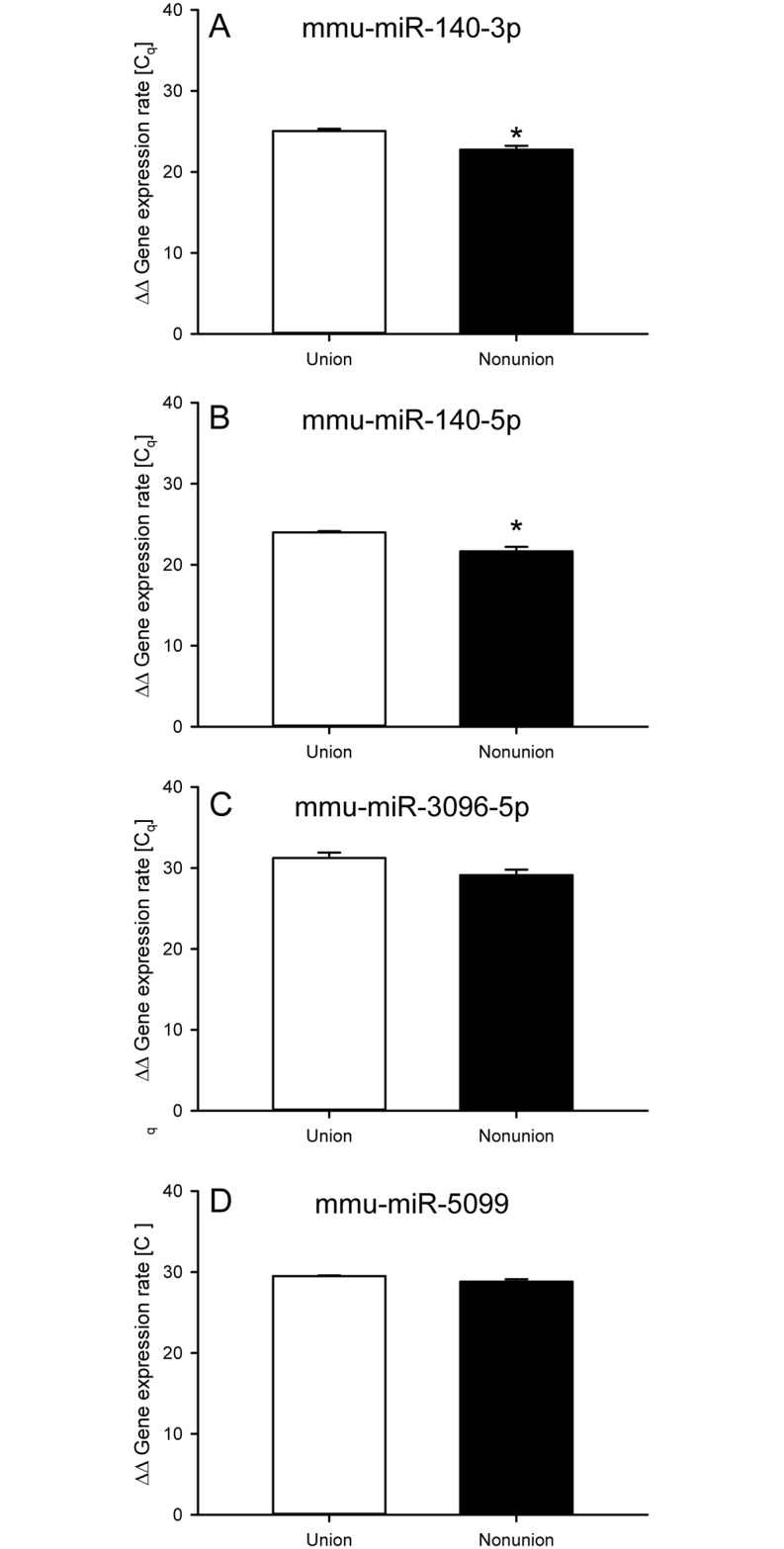
qrt-PCR analyses of callus tissue confirms different expression levels for miR-140-3p and miR-140-5p. **A-D:** ΔΔ Gene expression rate after qrt-PCR analyses of mmu-miR-140-3p (**A**), mmu-miR-140-5p (**B**), mmu-miR-3096-5p (**C**) and mmu-miR-5099 (**D**) at 7 days after surgery within the callus of the Union group (white bars; *n* = 4) and the Nonunion group (black bars; *n* = 4). Mean ± SEM; *p <0.05 vs. Union.

In the Nonunion group, qrt-PCR analyses of the selected miRs from the microarray analyses revealed a lower ΔΔ Gene expression rate for the miRs mmu-miR-140-3p and mmu-miR-140-5p (Fig 3A and 3B). Thus, the microarray results for these miRs could be validated, suggesting a higher expression of mmu-miR-140-3p and mmu-miR-140-5p within the callus tissue of nonunions. In contrast, qrt-PCR for the miRs mmu-miR-3096-5p and mmu-miR-5099 could not show significant differences in ΔΔ Gene expression rate (Fig 3C and 3D). This, in turn, indicates that mmu-miR-3096-5p and mmu-miR-5099 do not contribute substantially to nonunion formation.

### 3.4 Western blot analyses

**Fig 4:**
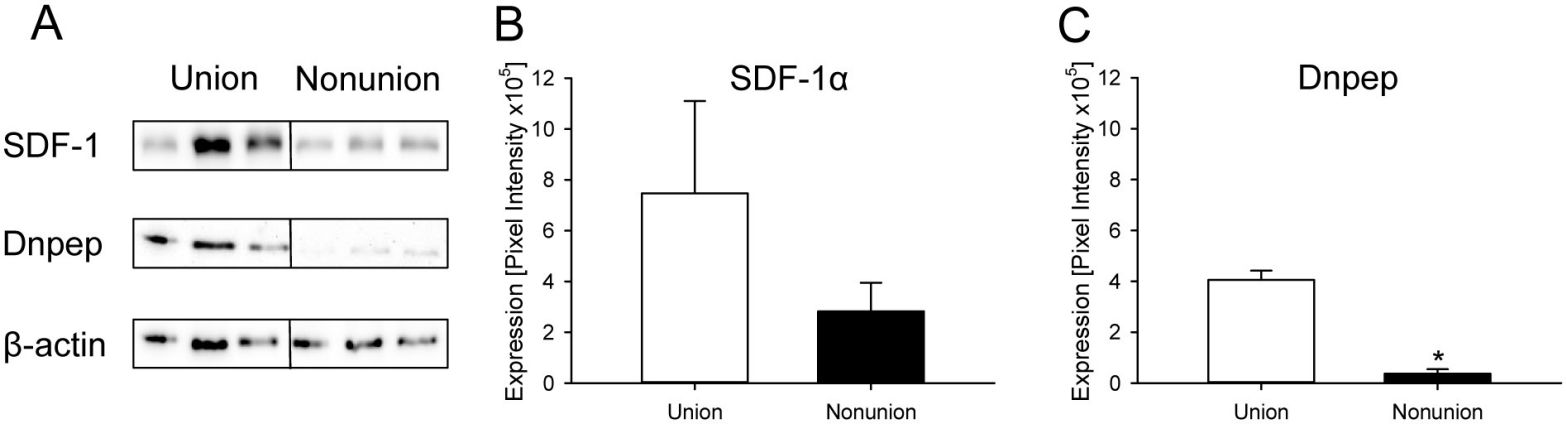
Western blot analyses of callus tissue shows different expression levels for target proteins of miR-140. **A:** Representative Western blots of SDF-1α, Dnpep, and β-actin expression within the callus tissue of the Union group and the Nonunion group at 7 days after osteotomy. **B, C:** Quantitative analysis of the expression of SDF-1α (**B**) and Dnpep (**C**) within the callus tissue of the Union group (white bars; *n* = 3) and the Nonunion group (black bars; *n* = 3) at 7 days after osteotomy. Mean ± SEM; *p < 0.05 vs. Union.

Western blotting of SDF-1α within callus tissue showed a markedly lower expression in the Nonunion group compared to the Union group (Fig 4A and 4B). In addition, the expression of Dnpep was significantly lower in the Nonunion group (Fig 4A and 4C).

## 4. Discussion

The present study indicates different expression patterns of miRs in callus tissue of nonunions compared to physiologically healing bones after osteotomy in mice. A total of 44 miRs were identified, which may be relevant for the bone healing process in mice. Nonunions revealed a higher expression level of mmu-miR-140-3p and mmu-miR-140-5p. This was associated with lower expression levels of SDF-1α and Dnpep, which are known to be target proteins of miR-140. These novel data suggest that the expression of mmu-miR-140-3p and mmu-miR-140-5p markedly contributes to the development of nonunions.

The process of bone healing represents a well-orchestrated series of biological events in vivo. Lately, a number of different bone healing models have been established to investigate the different mechanisms and molecular pathways of bone healing [25]. Each of these bone healing models shows differences in the grade of invasivity, complexity of the surgical procedure, mode of fracture and type of fracture model [25]. In the present study the ‘pin-clip’ model was used. This is an established bone defect model that enables the induction of physiological bone healing as well as nonunion formation by creating different gap sizes via the lateral approach. Although the healing response is known to result in either bone healing or nonunion formation, this response is only dependent on the different gap size and periosteum stripping, but not on other factors such as e.g. injury to the surrounding soft tissue. This makes the ‘pin-clip’ model a standardized bone healing model that allows the assessment of both, physiological bone healing and non-union formation. Besides, the cost for the use of this model are relatively low, especially when compared to the LockingMouseNail, which also allows the study of physiological healing and non-union formation in the mouse femur [25]. Therefore, we felt that using the ‘pin-clip’ model was suitable for the purpose of the present study.

The expression of miRs and proteins within the callus tissue was assessed at day 7 after osteotomy. At this early time point it cannot be seen if the osteotomy with the respective gap size will result in union or nonunion. However, a number of previous studies using this bone defect model has shown that a gap size of 1.8 mm with additional periosteum stripping results in nonunion formation, if the bone defect is not treated with angiogenic or osteogenic growth factors [11,13,26]. It is well known that during nonunion formation the callus composition changes markedly. While fully established nonunions after 70 days exhibit mainly fibrous tissue within the osteotomy gap, healing bones at this time point contain predominantly osseous tissue [11]. We are aware that in the present study fully established nonunions might have revealed more defined miR expression patterns compared to physiologically healing bones. However, at this late time point the differences in callus composition would have progressed much further. Given the fact that miR expression profiles are tissue-specific [27], we selected an early time point of the regeneration process in order to study a tissue composition which is comparable between the two groups. It is possible that tissue harvesting at even earlier time points than day 7 postoperatively may have revealed an even more comparable tissue composition. However, pilot studies have shown a very fragile callus especially in the Nonunion group before day 7, bearing the risk of non-standardized callus tissue harvesting.

In the course of murine bone healing the selected time point of 7 days represents the second phase of endochondral ossification. This is associated with the recruitment of fibroblasts, immune cells and stem cells, which all contribute to soft callus formation [12,28]. Therefore, the results of the Union group represent the expression pattern of miRs during the second phase of endochondral ossification in mice.

Of interest, remarkable differences in miR expression were observed between the two groups already at the early 7-day time point chosen. The Nonunion group exhibited a number of differently expressed miRs, as indicated by the microarray results. In fact, some of these miRs have been reported to be associated with bone formation and chondrogenesis, such as miR-181d, miR-140, miR-27a, miR-31, miR-214, miR-199 and miR-483 [29]. These findings indicate that the development of nonunions is not associated with a different expression of a single miR, but rather results from a distinct expression pattern of a variety of miRs.

Of interest, other miRs have also been reported to affect bone healing such as miR-29b [30]. Accordingly, application of miR-29b at 2 and 3 weeks after surgery improved bone repair [30]. However, in the present study no significant differences for this miR could be observed between the two study groups at day 7 after surgery. Therefore, particular miRs may be expressed at different stages throughout the healing process. This is in line with previous studies that revealed different expression levels for specific miRs during fracture healing in rats [18]. Thus, the expression of miRs over the time course of healing appears to be rather a dynamic process than a rigid pattern that is maintained throughout the entire regeneration process.

At 7 days after surgery both mmu-miR-140-3p and mmu-miR-140-5p revealed a significant difference between the two groups and showed a FC of >2.0. This difference could be validated by qrt-PCR. Thus, both forms of mmu-miR-140 seem to influence the healing process at this stage. In fact, miR-140-3p has previously been reported to negatively regulate the inflammatory signaling of nuclear factor-κB (NF-κB) and thereby acts as an antagonist to tumor necrosis factor (TNF)-α signalling [18,20,21]. Because inflammation and recruitment of inflammatory cells during this phase of bone healing is physiological, the increased levels of miR-140-3p in the Nonunion group of the present study may have contributed to the impaired healing by downregulating the inflammatory response.

Mmu-miR-140-5p has been reported to be abundantly expressed in cartilage tissue [22]. As demonstrated by Nakamura et al. [19], knockout of miR-140-5p results in defects during endochondral bone development. This effect is due to accelerated chondrocyte differentiation and, accordingly, an increased initial mineralization of various bones in miR-140-null mice [18,19]. While loss of miR-140-5p results in abnormalities during endochondral bone growth, it may be speculated that low miR-140-5p levels are beneficial for the healing process of injured bone, because the healing may benefit from the acceleration of chondrocyte differentiation and the increase of mineralization. Of interest, the results of the present study demonstrate an upregulation of miR-140-5p in animals of the Nonunion group. Accordingly, over-expression of miR-140-5p in these animals may contribute to an impairment of bone healing.

The increased levels of miR-140 were associated with a decreased expression of the proteins SDF-1α and Dnpep in animals of the Nonunion group compared to animals of the Union group. These proteins are known to be target proteins of miR-140 and have been reported to have key roles in the bone healing process [19,23]. SDF-1α, also known as CXC group of chemokine ligand 12 (Cxcl12), plays an important role in tissue repair in various organs. During bone healing, SDF-1α is indispensable for the biological effect of bone morphogenetic protein (BMP)-2 [31–33]. BMP-2 in turn has been shown to promote differentiation of mesenchymal stem cells into osteogenic lineages and has promoted bone repair in previous studies [13,34]. Accordingly, it may be speculated that nonunion formation may be due to an indirect inhibitory effect on BMP-2 through the downregulation of SDF-1α by miR-140.

Dnpep is known to antagonize the biological effect of BMP-2 [19]. In the present study, increased levels of miR-140 were associated with a reduced expression of Dnpep. We are aware that this association does by no means prove a causative relationship between changes of the miR-140 abundance and the reduced levels of Dnpep. The decrease of Dnpep may be interpreted as a physiological counter response to the reduced BMP-2 effect caused by the SDF-1α reduction. However, the indirect stimulation of BMP-2 by the decreased Dnpep expression in nonunions may not be sufficient to outweigh the inhibitory effect on SDF-1α.

We are aware that many other molecules and mechanisms, apart from miR-140, may potentially contribute to the process of nonunion formation and that the impact of increased miR-140 on non-union formation through decreased SDF-1α and Dnpep expression has to be investigated in more detail in further studies, using miR-140-5p-null mice, anti-miR-140-5p, and as control Mimics for miR-140-5p. Nonetheless, the present study describes for the first time an association between changes of miR-140 levels and changes of the levels of proteins Dnpep and SDF-1α as potentially contributing factors in the process of nonunion formation, which may open the door for novel treatment strategies.

The present study demonstrates that miR-140 plays a major role in the early stages of bone healing, however, the relevance of other miRs was not validated. Microarray results of miR-3096-5p and miR-5099 were examined by qrt-PCR and could not identify a relevant impact on bone healing. However, it cannot be excluded that other miRs may interfere with the actions of miR-140 and may also contribute to the process of bone healing.

## 5. Conclusions

In conclusion, the present study demonstrates different miR expression patterns for nonunions compared to physiological bone healing in mice during the early stage of bone healing. The present data further highlight a role of miR-140 in nonunion formation, most likely by inhibiting the inflammatory response and, indirectly, modulating the biological effect of BMP-2. Accordingly, further studies may elucidate whether a manipulation of miR-140 expression is effective in the prevention or treatment of nonunion formation.

## Supporting information

S1 File Results of microarray analyses. The microarray data of this publication have been deposited in NCBI’s Gene Expression Omnibus [35] and are accessible through GEO Series accession number GSE133243 (https://www.ncbi.nlm.nih.gov/geo/query/acc.cgi?acc=GSE133243).
(XLS)
